# A retrospective study of the efficacy of JAK inhibitors or abatacept on rheumatoid arthritis-interstitial lung disease

**DOI:** 10.1007/s10787-022-00936-w

**Published:** 2022-04-24

**Authors:** Marika Tardella, Marco Di Carlo, Marina Carotti, Luca Ceccarelli, Andrea Giovagnoni, Fausto Salaffi

**Affiliations:** 1 Rheumatology Clinic, Ospedale “Carlo Urbani”, Università Politecnica Delle Marche, Via Aldo Moro 25, 60035 Jesi, Ancona Italy; 2 Department of Radiology, Ospedali Riuniti, Università Politecnica Delle Marche, Ancona, Italy; 3 Department of Interventional and Diagnostic Radiology, Azienda Ospedaliero-Universitaria Sant’Anna, Ferrara, Italy

**Keywords:** Rheumatoid arthritis, Interstitial lung disease, Tofacitinib, Baricitinib, Abatacept, Janus-kinase inhibitors, High-resolution computed tomography

## Abstract

**Objectives:**

To examine the effectiveness of Janus-kinase inhibitors (JAKis) or abatacept (ABA) in patients with rheumatoid arthritis-interstitial lung disease (RA-ILD).

**Methods:**

Patients with RA-ILD receiving JAKis or ABA were retrospectively evaluated at baseline and after 18 months of treatment. A computer-aided method (CaM) was used to assess the extent of high-resolution computed tomography (HRCT) fibrosis percentage. According to HRCT fibrosis changes, patients were classified as “worsened” (progression of 15% or more), “stable” (changes within 15%) or “improved” (reduction of 15% or more). Correlations between RA characteristics and JAKis or ABA responses were studied using a multivariate regression model.

**Results:**

Seventy-five patients (69.3% women) were evaluated, 31 received a JAKi while 44 received ABA. In the JAKis group, five patients (16.1%) showed RA-ILD progression, 20 patients (64.5%) were considered stable, and six patients (19.4%) demonstrated RA-ILD improvement. In the ABA group, five patients (11.3%) showed RA-ILD progression, 32 patients (72.7%) were stable, and seven patients (16.0%) demonstrated RA-ILD improvement. In both groups, the percentage of current smokers was different between those classified as "worsened" and those classified as "improved/stable" (*p* = 0.01). In multivariate regression analysis, current smoking habit (*p* = 0.0051) and concomitant methotrexate treatment (*p* = 0.0078) were the two variables related to RA-ILD progression in ABA-treated patients, whereas in JAKis-treated patients, the only RA-ILD progression-related variable was disease duration of RA (*p* < 0.001).

**Conclusions:**

Treatment with JAKis or ABA was related to stability or improvement of RA-ILD in 83.9% and 88.6% of patients, respectively. RA duration is the only variable associated with worsening RA-ILD in JAKis-treated patients.

## Introduction

Rheumatoid arthritis (RA) is a chronic autoimmune disease characterized by joint inflammation and destruction, with an incidence of 0.5% of the adult population in Western countries (Salaffi et al. 2005). In addition to joint involvement, extra-articular manifestations of RA can affect several organs and systems. Pulmonary involvement in RA has been the subject of increasing interest in recent years, particularly interstitial lung disease (ILD) as one of the most severe extra-articular manifestation, leading to progressive respiratory failure (Salaffi et al. 2019). ILD occurs in 1.8% to 67% of RA patients, determining a worse prognosis and mortality (Fazeli et al. 2021; Spagnolo et al. 2018; Raimundo et al. 2019). The management and treatment of RA-ILD are challenging because there is still little information available and there are no clinical trials dedicated on the topic, although this aspect is increasingly considered by guidelines (Holroyd et al. 2019). Current therapies for RA treatment are worldwide extensively evaluated to determine their effect on lung and RA-ILD patients. Since few years the efficacy of anti-fibrotic drugs used in idiopathic pulmonary fibrosis has been evaluated in RA-ILD patients (Redente et al. 2018). Many authors discuss the lung toxicity of disease modifying anti-rheumatic drugs (DMARDs), particularly methotrexate (England and Hershberger 2020; Roubille and Haraoui 2014). If until recently methotrexate was thought to be toxic for patients with RA-ILD because it could lead to pulmonary toxicity, now the most recent data show that it could slow down the progression of lung damage (Wells 2021; Rojas-Serrano et al. 2017).

The introduction of biotechnological DMARDs (bDMARDs) and, more recently, of Janus-kinase inhibitors (JAKis) has changed the course of RA, markedly improving the control of synovitis and, consequently, reducing joint destruction and physical disability (Nash et al. 2021; Fraenkel et al. 2021). We have recently underlined the “protective” effect of abatacept (ABA), a T lymphocyte co-stimulation antagonist, in patients with RA-ILD, showing reasonable efficacy in slowing the progression of RA-ILD in about 88% of cases (Tardella et al. 2021). Other bDMARDs are suggested for RA-ILD therapy, such as rituximab and tocilizumab, but in addition to their higher infectious risk compared with ABA, cases of onset or worsening of ILD have been reported in patients taking these drugs (Manfredi et al. 2020; Soubrier et al. 2008; Wendling et al. 2013; Hadjinicolaou et al. 2011).

JAKis is a group of drugs classified as "small molecules" or "targeted synthetic DMARDs" to differentiate themselves from bDMARDS by the different mechanism of action. They are medications that are taken orally every day and have demonstrated efficacy in the treatment of RA, either alone or in combination with methotrexate (Fleischmann et al. 2017; Lee and Bae 2016). Little is known about the safety and tolerability of JAKis in patients with RA-ILD (Khoo et al. 2020; Salvarani et al. 2021; Citera et al.  2021). New data are emerging in favor of tofacitinib (TFN), the longest used JAKi, to slowing the progression of ILD associated to connective tissue diseases (Romero-Bueno et al. 2020; Chen et al. 2019; Pineton De Chambrun et al. 2020). Sendo and colleagues showed the effect of TFN on mice that developed arthritis and ILD, revealing a significant slowdown in ILD progression (Sendo et al. 2019). Lescoat and colleagues tested the effect of ruxolitinib, a JAKi used in the treatment of myelofibrosis, on mice with scleroderma-like ILD, revealing improved skin and lung involvement (Lescoat et al. 2020). These preliminary data prompt testing of the combined anti-inflammatory and anti-fibrotic properties of JAKis also in patients with RA-ILD.

The primary outcome of interest of this study is the evaluation of the evolution of pulmonary fibrosis in patients receiving JAKis compared to those receiving ABA, using computerized HRCT assessment. A secondary objective is to identify predictors of an unfavorable treatment outcome of RA-ILD during JAKis therapy.

## Materials and methods

### Study population and assessment

Study data were extracted from a database dedicated to RA patients referred to the Rheumatology Clinic, Università Politecnica delle Marche (Italy). Patients were included if aged > 18 years, met the American College of Rheumatology (ACR)/EUropean League Against Rheumatism (EULAR) classification criteria for RA diagnosis and, concomitantly, the American Thoracic Society/ATS/ERS 2015 criteria for ILD diagnosis (Aletaha et al. 2010; Travis et al. 2013). Moreover, patients included in the study were: RA-ILD patients who took continuous JAKi therapy (TFN at an oral dose of 5 mg BID or baricitinib at an oral dose of 4 mg daily) or ABA therapy at a standard dose of 125 mg/week subcutaneously for at least 18 months, who performed a rheumatologic evaluation [tender joint count (TJC) and swollen joint count (SJC), Clinical Disease Activity Index (CDAI), Health Assessment Questionnaire-Disability Index (HAQ-DI)] and a pulmonary investigation [high-resolution computed tomography of the lung (HRCT), pulmonary function test (PFT), single-breath diffusion lung capacity of carbon monoxide (DLco, % predicted, corrected for hemoglobin), Borg's dyspnea index (BDI)] within two weeks the beginning of a JAKi or ABA (time 0) and after 18 months (time 18). We included patients with ≥ 10% extent of fibrosis on HRCT. We considered the group of patients treated with ABA as a control group because it is one of the recommended treatments in RA-ILD. Patients concomitantly taking methotrexate (MTX) or other conventional synthetic DMARDs (csDMARDs) and/or glucocorticoids at a dose of less than 10 mg daily prednisone or equivalent were included. Patients with a history of lung diseases, other than ILD, and/or NYHA stage II-IV heart failure and patients who had been previously treated with ABA or JAKis were excluded.

Baseline data included demographic variables, smoking habits, duration of disease (defined as time elapsed since diagnosis), and the presence or absence of rheumatoid factor (RF) and anti-citrullinated peptide antibodies (ACPA).

The evaluation of the ILD extension was performed with a computer-aided method (CaM) able to estimate the percentage of fibrosis at HRCT, based on what has been described in detail in previous works (Salaffi et al. 2020a; Ariani et al. 2014; Salaffi et al. 2020b). The CaM was performed with OsiriX MD 7, a DICOM visualization software (OsiriX MD version 7, 64-bit format) on a Mac Mini (2.8 GHz Intel Core 2 Duo Desktop Computer, 16 GB random access memory; Apple Computer, Cupertino, CA, USA) with Mac OSX 10.12.2 operating system. Two independent radiologists, blind to the clinical data, evaluated HRCT lung abnormalities and the CaM quantification process.

According to HRCT results, performed at time 0 and time 18, patients were divided into three groups based on HRCT-CaM comparison: the "worsened" group included patients with ≥ 15% progression of pulmonary fibrosis, the "improved" group included patients with ≥ 15% reduction of pulmonary fibrosis, and the "stable" group included patients with progression or reduction of fibrosis < 15%. The 15% CaM-change threshold derived from the standard deviation of the change in mean value after 18 months of follow-up.

### Statistical analysis

Data were processed with MedCalc 19.0.6 (statistical software packages for Windows XP). Normal distribution was tested using the Kolmogorov–Smirnov test. The median and interquartile ranges (IQR) as well as the mean and standard deviations (SD) were presented wherever appropriate. Continuous variables were compared using the parametric two-sample *t* test and the one-way analysis of variance (ANOVA) test, while categorical variables among patients were compared using the χ^2^ test. Values at time 0 and time 18 were compared with the two-sided coupled *t* test and the non-parametric Wilcoxon signed rank test.

Corrected multivariate regression analysis was used to assess the strength of the association between RA characteristics and HRCT response to ABA or JAKis, considering CaM quantification as a dependent variable. Age, sex, disease duration, disease onset, smoking habit, presence of RF, presence of ACPA, CDAI, and HAQ-DI were the covariates. Results were expressed as multivariate regression coefficient (R) and adjusted quadratic regression coefficient (R2) for the number of variables included in the analysis. Significance was set at *p* < 0.05.

## Results

We enrolled 75 patients (69.3% women, mean age 59.5 ± 7.77 and mean disease duration 7.44 ± 3.25 years), of whom 31 received a JAKi (18 patients took baricitinib, 13 patients took TFN) and 44 were treated with ABA. Both treatment groups had similar demographic and clinical features (Table 1). As shown, at time 0 the included patients had on average a mild to moderate lung function impairment (forced vital capacity 81.7% and DLco 59.2%) and the CaM assessment showed a mean fibrosis rate of 19.4 and 18.5% in patients treated with ABA or JAKis, respectively. A fibrosis rate greater than 20% at CaM evaluation occurred in 14/31 (45%) and 18/44 (41%) of patients in the JAKis and ABA groups, respectively. In ABA group 23 (52.3%) patients were ACPA positive and 28 (63.6%) RF positive, all patients were concomitantly treated with csDMARDs, 16 (36.4%) patients were previously treated with a bDMARD and 31 (70.4%) patients took steroids at a mean dose of 3.7 (range 1.25–8.5) mg prednisolone/day equivalent. In JAKis group 16 (51.6%) patients were ACPA positive and 19 (61.3%) RF positive, no patients were simultaneously taking csDMARDs, 11 (35.5%) patients were previously treated with a bDMARD and 21 (67.7%) patients took steroids at a mean dose of 3.3 (range 1.11–8.22) mg prednisolone/day equivalent.

**Table 1:** Clinical and lung functional data, and significance level at abatacept and JAKis beginning (Time 0), and after 18 months of treatment (Time 18), expressed in means and standard deviations

	Abatacept			JAKis		
	Time 0	T18	Significance level (*p*)*	Time 0	Time 18	Significance level (*p*)*
Age (years)	59.05 ± 8.03	–	–	59.87 ± 7.52	–	–
Disease duration (years)	7.55 ± 3.09	–	–	7.33 ± 3.41	–	–
CDAI	34.66 ± 10.05	10.11 ± 7.58	< 0.001	38.56 ± 9.47	8.77 ± 7.24	< 0.001
HAQ-DI	1.45 ± 0.32	0.75 ± 0.29	< 0.001	1.54 ± 0.36	0.71 ± 0.28	< 0.001
Borg Dyspnea Index	2.54 ± 1.23	1.90 ± 1.01	0.01	2.51 ± 1.22	1.87 ± 1.11	0.03
DLco (% predicted)	58.69 ± 8.24	61.26 ± 11.23	0.22	59.72 ± 8.56	62.75 ± 11.84	0.28
FVC (% predicted)	82.29 ± 4.86	81.24 ± 11.97	0.59	81.18 ± 5.07	79.59 ± 14.02	0.55
HRCT-CaM fibrosis (%)	19.41 ± 5.89	18.94 ± 6.06	0.71	18.54 ± 6.31	17.52 ± 6.35	0.53

*CDAI* Clinical Disease Activity Index, *HAQ-DI* Health Assessment Questionnaire Disability Index, *DLco* diffusion lung capacity of carbon monoxide, *FVC* forced vital capacity, *HRCT* high-resolution computed tomography, *CaM* computer-aided method

^*^two-sided paired Student *t* test

**Fig. 1:**
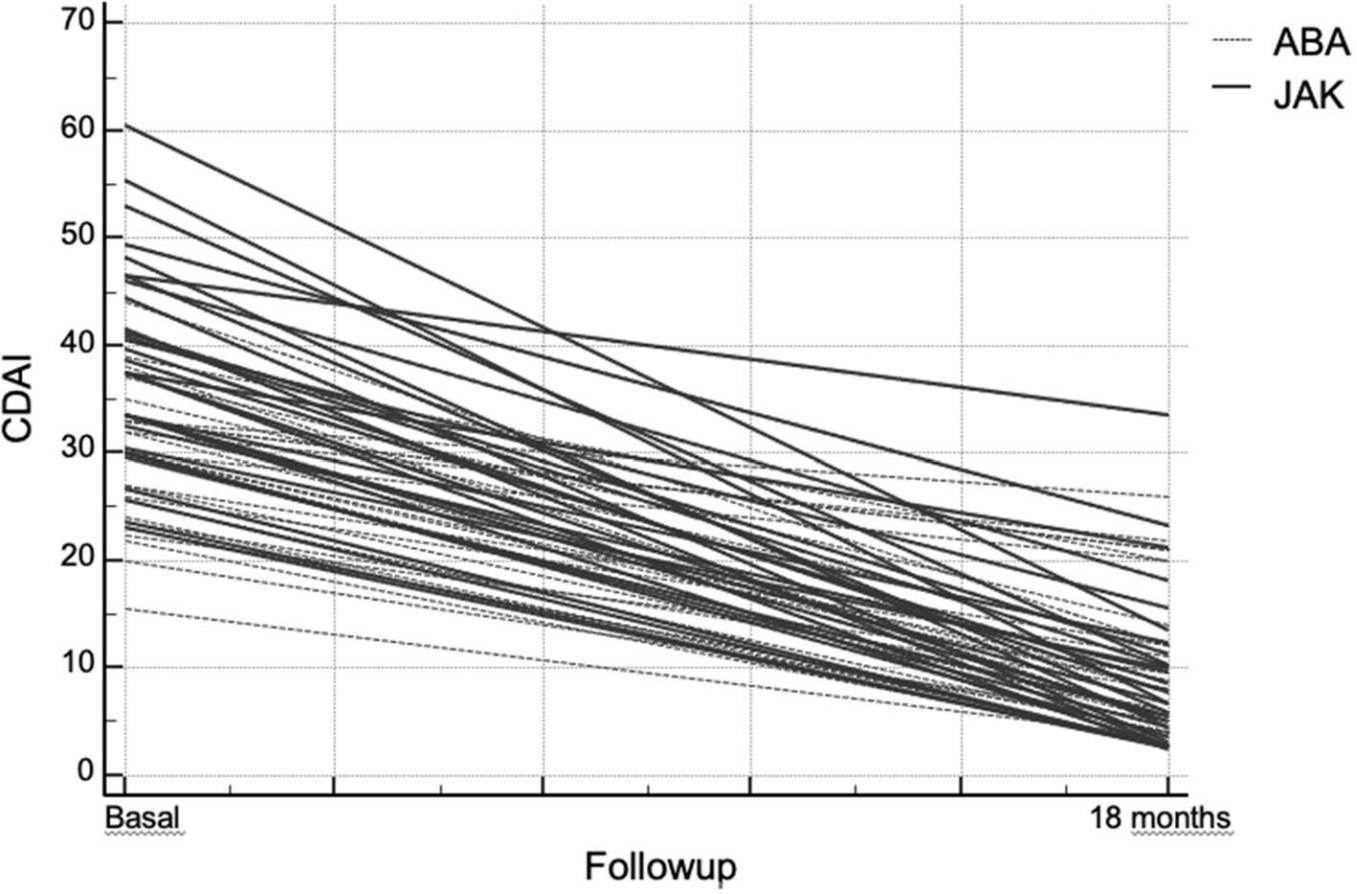
Trend in disease activity (Clinical Disease Activity Index) after 18 months of treatment with Janus-kinase inhibitors or abatacept. *ABA* abatacept, *CDAI* Clinical Disease Activity Index, *JAK* janus-kinase inhibitors

**Fig. 2:**
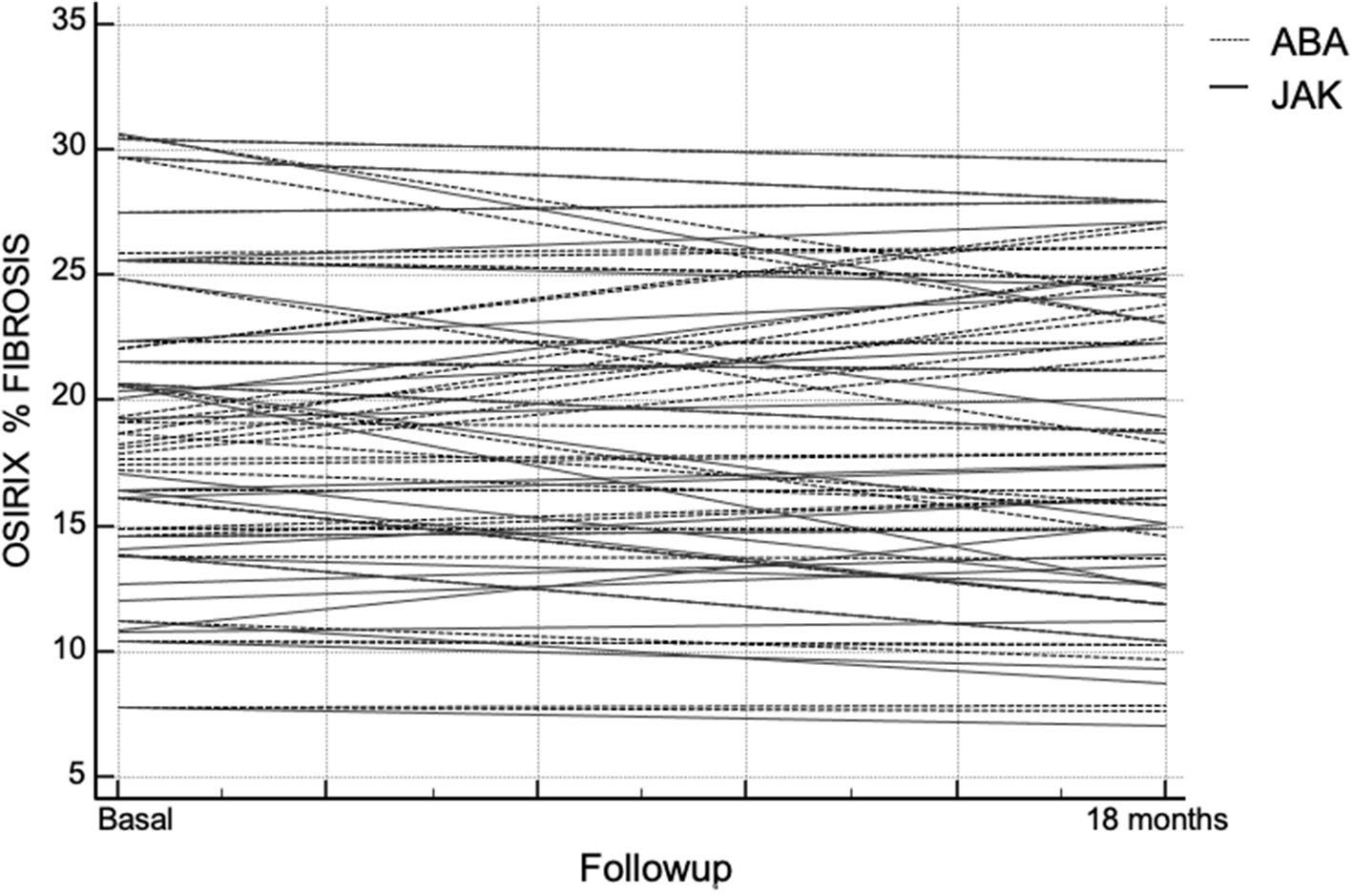
Trend in lung fibrosis rate (estimated by OsiriX, computer-aided method) after 18 months of treatment with Janus-kinase inhibitors or abatacept *ABA* abatacept, *JAK* janus-kinase inhibitors

At time 18 the RA articular manifestations had a statistically significant improvement (*p* < 0.001) both as an estimate of disease activity and as of patients-outcome in both treatment groups (Fig. 1). In contrast, the pulmonary component did not show a statistically significant reduction for both patients-outcomes and instrumental evaluation (Fig. 2). In ABA group 5 (11.4%) patients showed a HRCT deterioration, 32 (72.6%) were considered stable, 7 (16.0%) patients showed an HRCT improvement, while in JAKis group 5 (16.1%) patients showed a HRCT deterioration, 20 (64.5%) were considered stable, 6 (19.4%) patients showed an HRCT improvement at time 18.

Considering the whole cohort, the percentage of current smokers was different between those classified as "worsened" and those classified as "improved/stable" (*p* = 0.01).

In multivariate regression analysis, current smoking habit (*p* = 0.005) and concomitant methotrexate treatment (*p* = 0.0078) were the two variables related to RA-ILD progression in ABA-treated patients. In JAKis-treated patients, the only variable related to RA-ILD deterioration was disease duration (*p* < 0.001), whereas smoking exposure, immunological profile, lung function, percentage of lung fibrosis at time 0, and disease activity did not show a statistical correlation (Table 2).

**Table 2:** Multivariate regression analysis of the variables predictive of pulmonary fibrosis evaluated at high-resolution computed tomography by the computer-aided method (dependent variable) in patients treated with Jak inhibitors

Independent variables	Coefficient	Standard error	T	*p*	*r* partial
(Constant)	25.9222				
Age (years)	− 0.08694	0.1064	− 0.817	0.4176	− 0.1137
Gender	0.7888	1.5649	0.504	0.6164	0.07041
Disease duration (years)	1.0832	0.2016	5.372	0.0010	0.6011
ACPA positivity	− 2.4294	1.3316	− 1.824	0.0740	− 0,2475
RF positivity	2.0201	1.4028	1.440	0.1560	0.1977
Current smokers	− 1.9537	1.1232	− 1.112	0.2112	− 0.2321
DLco (% predicted)	− 0.1534	0.07260	− 2.113	0.0595	− 0.2837
FVC (% predicted)	− 0.04102	0.06492	− 0.632	0.5303	− 0.08813
CDAI	0.05518	0.03985	1.385	0.1722	0.1904
HAQ-DI	0.003992	0.02563	0.156	0.8768	0.02181

*ACPA* anti-cytrullinated protein antibodies, *RF* rheumatoid factor, *DLco* diffusion lung capacity of carbon monoxide, *FVC* forced vital capacity, *CDAI* Clinical Disease Activity Index, *HAQ-DI* Health Assessment Questionnaire Disability Index

## Discussion

ILD is an insidious condition in terms of morbidity and quality of life in patients with connective tissue diseases. Our study highlights the efficacy of JAKis in patients with RA-ILD, in relation also to the results obtained in a similar cohort treated with ABA. To the best of our knowledge, this is the first study evaluating the efficacy of JAKis treatment in patients with RA-ILD by assessing the evolution of lung fibrosis rate with a HRCT-CaM. In 83.9% of RA-ILD patients on JAKis therapy there was no deterioration of the lung disease. This finding is like that obtained in RA-ILD patients treated with ABA (88.6%), representing another step in the knowledge of the complex RA-ILD therapy.

As mentioned, there are encouraging efficacy data of JAKis in studies performed in RA-ILD mice and, especially, in patients with RA-ILD-related myositis (Chen et al. 2019; Citera et al. 2021; Khoo et al 2020; Romero-Bueno et al. 2020; Salvarani et al. 2021). Data on the efficacy of JAKis in the treatment of RA-ILD have not yet been published, except for a few case reports or studies with small groups of patients. Saldarriaga-River and colleagues reported the use of tofacitinib in three RA patients, two with ILD and one with chronic obstructive pulmonary disease. After 8–12 months, they had no lung disease exacerbations and hospitalizations, achieving good control of joint disease (Saldarriaga-Rivera and López-Villegas 2019). A recent Italian monocentric study investigated the efficacy of baricitinib in 11 RA and 4 RA-ILD patients for 6 months, analyzing the trend of serum values of proinflammatory cytokine and lung function test parameters. They found an improvement in DLco and diffusion coefficient (KCO) percentages during baricitinib treatment in the entire population. They also noted a reduction in KL-6, a molecule produced predominantly in the lung by damaged type II pneumocytes, but they did not evaluate the evolution of pulmonary fibrosis at HRCT (d’Alessandro et al. 2020). Another case of a patient with progressive RA-ILD treated with TFN was recently published, showing stabilization of ILD and improvement of respiratory symptoms. The authors emphasized the advantage of managing JAKis in case of infectious complications due to its short half-life (Vacchi et al. 2021). This aspect is not minor because respiratory tract infections in patients treated with JAKis are frequent and insidious. We were unable to study safety data because there was a lack of available data. Salvarani and colleagues recently estimated the incidence of infectious and non-infectious ILD in baricitinib-treated RA patients. They performed a review of 3770 patients with RA from eight randomized clinical trials and one long-term extension study on baricitinib, concluding that RA patients treated with baricitinib were at low risk of developing ILD (Salvarani et al. 2021). A similar analysis was performed on 21 tofacitinib clinical trials, in which 42 (0.16%) ILD events were identified among 7061 RA patients, confirming the low risk of developing ILD in JAKis-treated patients. The authors found also that age 65 years or older, current smokers, and a high disease activity index are the risk factors associated with the development of RA-ILD (Citera et al. 2021). In our analysis, the only variable related to ILD progression in JAKis group was RA disease duration while the remaining covariates did not show correlation, and this could be due to the small number of patients enrolled or a bias in recruitment. This aspect is of non-unique interpretation and in many studies on the topic the results are not always concordant (Dawson et al. 2002; Zamora-Legoff et al. 2017). However, disease duration is one of the already known risk factors to be considered in the management of RA patients.

In 2014 two new anti-fibrotic drugs (nintedanib and pirfenidone) were licensed to treat idiopathic pulmonary fibrosis. Data on their efficacy in the treatment of nonidiopathic ILD are emerging. Data from the INBUILD trial show the efficacy of nintedanib, a small molecule protein kinase inhibitor, in patients with ILD in connective tissue diseases. Eighty-nine RA patients, 44 patients with systemic sclerosis and 20 patients with mixed connective tissue disease were recruited in the study (Wells et al. 2020). The drug reduced the annual rate of decline in forced vital capacity by 57% compared to placebo, promoting drug approval by the US Food and Drug Administration for progressive-ILD, including connective tissue disease-ILD. Additional data are emerging from real-life studies (Vacchi et al. 2020). Narvaez and colleagues used nintedanib in combination with immunosuppressants for at least 6 months in six RA-ILD patients. They showed a reversal of the decline in lung function parameters, achieving a stabilization of forced vital capacity and DLco (Narváez et al. 2020). In RA-ILD mice models pirfenidone has demonstrated an inhibitory effect on transition from fibroblast to myofibroblast in the lungs (Wu et al. 2019). At present a trial on safety and efficacy of pirfenidone on RA-ILD patients is ongoing (Solomon et al. 2019). Certainly, anti-fibrotic drugs offer a very important therapeutic opportunity in progressive RA-ILD as adjunctive therapy to DMARDs. However, it could lead to additional side effects (liver toxicity and diarrhea) reducing adherence to treatment. Therefore, it would be advisable using a drug that can be effective on both articular and extra-articular manifestations and JAKis might be the best choice.

In our study the progression of pulmonary fibrosis at HRCT is used to assess response to treatment. In literature, the most widely used method for this purpose is the trend of PFT parameters, particularly forced vital capacity, and of DLco. We use HRCT-CaM in daily practice because it is a reliable, reproducible and easily comparable parameter, so it might also be used in clinical research (Tardella et al. 2021, Salaffi et al. 2020b). PFT and DLco could be influenced by many factors, both pulmonary and extra-pulmonary, and are operator- and patient-dependent. This aspect is a distinctive feature of our research and is a hot topic in rheumatology investigation because of the clear advantages demonstrated, particularly the rapidity of estimating fibrosis percentage (Salaffi et al. 2020b).

This study has some limitations. It is a retrospective, single-center study with a small number of patients enrolled. We do not know how many patients had severe infectious complications that had to be permanently discontinued JAKis or ABA treatment. Furthermore, we do not know the onset of ILD so we do not know if they are progressive ILDs and the rate at which they are advancing. In addition, lung fibrosis in our cohort is mild to moderate with less than 20% fibrosis in 43/75 (57.3%) cases, so our results cannot be generalized to patients with severe forms of RA-ILD, which are the most difficult to manage in clinical practice, as they can lead to respiratory failure or death.

## Conclusions

Our study showed that JAKis-treated patients worsened in only 16% of cases. Therefore, it can be stated that JAKis are effective in slowing down fibrosis in RA-ILD, and they should be considered as a first-choice therapy in RA patients with active synovitis and ILD before a stage of extensive fibrosis. From these data, prospective studies with a larger cohort are mandatory to consolidate these promising results.

## Data Availability

The datasets generated during and/or analyzed during the current study are available from the corresponding author on reasonable request.
